# Protein Kinases and Phosphatases in the Control of Cell Fate

**DOI:** 10.4061/2011/329098

**Published:** 2011-09-04

**Authors:** Angela Bononi, Chiara Agnoletto, Elena De Marchi, Saverio Marchi, Simone Patergnani, Massimo Bonora, Carlotta Giorgi, Sonia Missiroli, Federica Poletti, Alessandro Rimessi, Paolo Pinton

**Affiliations:** Section of General Pathology, Department of Experimental and Diagnostic Medicine, Interdisciplinary Center for the Study of Inflammation (ICSI) and LTTA Center, University of Ferrara, 44100 Ferrara, Italy

## Abstract

Protein phosphorylation controls many aspects of cell fate and is often deregulated in pathological conditions. Several recent findings have provided an intriguing insight into the spatial regulation of protein phosphorylation across different subcellular compartments and how this can be finely orchestrated by specific kinases and phosphatases. In this review, the focus will be placed on (i) the phosphoinositide 3-kinase (PI3K) pathway, specifically on the kinases Akt and mTOR and on the phosphatases PP2a and PTEN, and on (ii) the PKC family of serine/threonine kinases. We will look at general aspects of cell physiology controlled by these kinases and phosphatases, highlighting the signalling pathways that drive cell division, proliferation, and apoptosis.

## 1. Introduction

Cells are exposed to several extracellular signals simultaneously. Maintaining the fidelity of intracellular transduction systems, resulting in the amplification of specific biological responses, is crucial in eliciting the appropriate physiological response. The accurate selection of effector molecules is required, and these molecules must be finely regulated in their activation and deactivation, often by phosphorylation and dephosphorylation events. Protein phosphorylation is a reversible posttranslational modification that plays key roles in several physiological processes and is often deregulated in pathological conditions. The balance between activation and deactivation of signalling pathways is a delicate one, which is regulated not only through phosphorylation by kinases, but also through dephosphorylation events induced by a diverse range of phosphatases. The majority of oncogenes identified thus far encode protein kinases, and dysregulation in their activity is required for cancer initiation and maintenance. Intuitively, by counterbalancing the activity of kinases, phosphatases should primarily act as tumour suppressors [1, 2]. Approximately one-third of proteins encoded by the human genome are presumed to be phosphorylated during their life cycle, accounting for an estimated 100,000 different phosphorylation sites in a cellular proteome [3]. The structural changes imparted by the phosphorylation of specific residues afford exquisite mechanisms for the regulation of protein functions by modulating protein folding, substrate affinity, stability, and activity. In many cases, phosphorylation results in switch-like changes in protein function, which can also bear to major modifications, that is, in the catalytic function of other enzymes, including kinases. Moreover, protein phosphorylation often leads to a structural change of the protein that can induce changes in interaction partners or subcellular localization.

Phosphorylation acts as a molecular switch for many regulatory events in signalling pathways that drive cell division, proliferation, differentiation, and apoptosis. One of the main strategies for achieving the proper outcome is the compartmentalization of both protein kinases and phosphatases, to ensure an appropriate balance of protein phosphorylation [4]. The spatial distribution of kinases and phosphatases implies that a gradient of phosphorylated substrates exists across different subcellular compartments. This spatial separation not only regulates protein phosphorylation but can also control the activity of other proteins, enzymes, and the transfer of other posttranslational modifications. The most important routes showing the fundamental importance of subcellular localization of kinases and phosphatases are perhaps the mitogen-activated protein-kinase- (MAPK-) mediated pathways, the phosphoinositide 3-kinase (PI3K)/Akt/mammalian target of rapamycin (mTOR)-dependent signalling, as well as those involving protein kinase A (PKA) and protein kinase C (PKC), both of which act as key transducers in many signalling cascades. These signalling pathways (MAPK, PI3K/Akt/mTOR, PKA, PKC) interact at numerous levels and at multiple intracellular sites to regulate many fundamental cellular processes. Crosstalk between signalling pathways is a common theme in cell regulation, which usually depends on cell context and plays an important role in fine-tuning the biological responses. It is now well established that MAPK and PI3K are two of the most predominant oncogenic routes, and they are intimately linked together [5, 6]. The signal transduction ensuing from these pathways is complicated by a remarkable number of interconnections [7] (Ras-MAPK pathway modulates PI3K pathway at multiple levels: MAPK—c-Jun—PTEN, Ras—PI3K, ERK—TSC2, RSK—TSC2, RSK—S6, RSK—eIF4B, and so does MKK4—JNK pathway through the activation of NF-*κ*B; for a recent review see [8]). Both MAPK and PI3K pathways may result in the phosphorylation of many downstream targets and impose a role in the regulation of cell survival and proliferation. Overexpression of these pathways in acute myeloid leukemias (AML) has been associated with a worse prognosis than overexpression of a single pathway [9], while activation of the MAPK (Raf/MEK/ERK) cascade is suppressed in some prostate cancer cell lines which express high levels of activated Akt [10, 11]. Hyperactivation of Akt has been found in cellular models of prostate cancer, such as LNCaP cells, as well as in prostate cancer specimens, particularly in advanced stages of the disease [12]. It has been demonstrated that in prostate cancer, PI3K-Akt survival pathway could be affected also by PKC activation. Indeed, PKC promotes apoptosis in LNCaP cells through activation of p38 MAPK, and inhibition of the Akt survival pathway [13]. Also PKA can act as a central hub that interacts with a variety of other signaling pathways, not only mediating but also communicating cAMP effects to Akt, PKC, MAPK and other pathways [14]. Recent studies indicated that cAMP-dependent signalling is closely interwoven with the PI3K/Akt pathway [15]. Akt is of tremendous importance for several neuronal key signalling events, including cell differentiation, proliferation, and survival [16]. Neuronal survival and axonal regeneration mediated by PI3K-dependent Akt signalling were shown to be induced by elevated cAMP levels [15, 17]. Finally, it is important to state that several of these major protein kinases in the cell, in particular Akt, PKC, and ERK MAP kinases, are substrates for the protein phosphatase 2a (PP2a), which appears to be the major phosphatase in eukaryotic cells that downregulates activated protein kinases. Thus, PP2a is likely to play an important role in determining the activation of protein kinase cascades [18].

**Figure 1:**
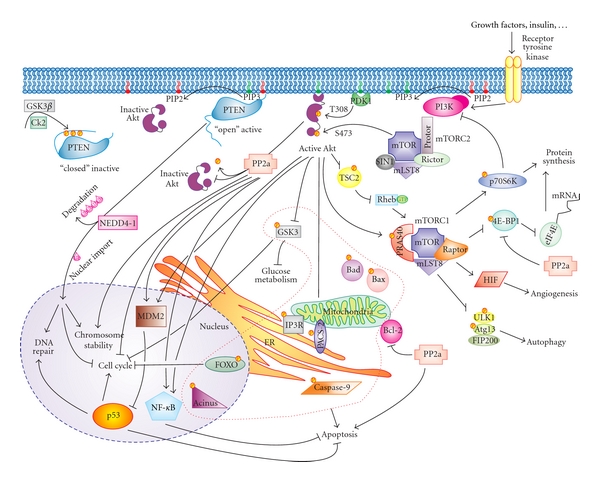
PI3K signalling pathway: The phosphatidylinositol 3-kinase (PI3K) signalling pathway begins with PI3K activation by receptor tyrosine kinases after growth factors or insulin stimulation. PI3K activity phosphorylates and converts the lipid second messenger phosphatidylinositol 4,5-bisphosphate (PIP2—indicated by red phosphoinositide) into phosphatidylinositol 3,4,5-triphosphate (PIP3—indicated by green phosphoinositide), with consequent double phosphorylation/activation of Akt kinase. Akt promotes cell proliferation and survival by phosphorylation/inhibition of several proapoptotic targets (hold by the red dotted line), or by activation of mTOR complex 1, an important regulator of several processes, such as autophagy, angiogenesis and protein synthesis. Protein phosphatase 2a (PP2a) family members are able to dephosphorylate/inhibit Akt, favouring apoptosis also by direct dephosphorylation of Bcl-2. The tumour suppressor phosphatase PTEN negatively regulates PI3K signalling by dephosphorylating PIP3, converting it back to PIP2. A mono-uniquitinated/active form of PTEN is able to translocate into the nucleus, promoting DNA repair, cell cycle arrest and chromosome stability.

In the first part of this paper we will focus on the PI3K/Akt/mTOR pathway, specifically on the kinases Akt and mTOR, along with the phosphatases PP2a and PTEN (phosphatase and tensin homolog deleted on chromosome 10). The PI3K/Akt/mTOR signalling cascade is crucial to many widely divergent physiological processes which include cell cycle progression, transcription, translation, differentiation, apoptosis, motility, and metabolism [19]. After the p53 pathway, the PI3K/Akt/mTOR signalling pathway is one of the most mutated pathways associated with human tumour and contributes to both cancer pathogenesis and therapy resistance [20]. The binding of insulin, insulin-like growth factor-1 (IGF-1), and other growth factors to its related receptors can activate/phosphorylate PI3K, which catalyses the synthesis of the lipid phosphatidylinositol 3,4,5-trisphosphate (PIP3). These lipid products in turn interact with proteins via their pleckstrin homology (PH) domain, which allows the recruitment of other signalling molecules to the cell membrane [21]. Akt and phosphoinositide-dependent kinase-1 (PDK1) are PI3K's main downstream effectors: they contain a C-terminal PH domain, which binds the membrane-bound PIP3. Here, Akt is activated through phosphorylation mediated by PDK1 [22] and by the mammalian target of rapamycin complex 2 (mTORC2) [23]. In turn, activated Akt phosphorylates many target proteins to regulate a broad range of cellular processes which include cell survival, growth, proliferation, angiogenesis, metabolism, and migration. This pathway is activated in many tumours as a result of amplification/overexpression of PI3K (either PI3CA or the p85 subunit) [24], or of the amplification/overexpression/mutation of Akt [25]. Moreover, a tight counterregulation by phosphatases has emerged as a crucial process to control PI3K/Akt/mTOR-dependent signalling. Elevated Akt activation in human cancers can result from its enhanced phosphorylation due to loss of the PTEN tumour suppressor [26] or PP2a, which is now considered to be a tumour suppressor as well since it can also dephosphorylate Akt and thus downregulate its activity [27]. Evidence accumulated over the past years has highlighted the presence of different players of this intricate pathway in almost all the different subcellular compartments (namely, cytosol, plasma membrane, nucleus, mitochondria, endoplasmic reticulum (ER) and mitochondrial-associated membranes (MAMs)), where they can both modulate each other and act on a plethora of different substrates (Figure 1). In the following sections, we will discuss these events and how they are implicated in the control of cell fate.

In the final part, we will address more in depth the PKC family of serine/threonine kinases that, when activated, can be translocated from one intracellular compartment to another, thus being able to affect a wide variety of cellular processes. PKC is one of the most extensively studied kinase families and has been implicated in cell proliferation, differentiation, apoptosis, tumour promotion, and neuronal activity [28]. There are 12 isoforms of PKC termed conventional (PKC*α*, PKC*β* and PKC*γ*), novel (PKC*δ*, PKC*ε*, PKC*η* and PKC*θ*), atypical (PKC*ζ* and PKC*λ*), and PKN and PKC-related (PKN1, PKN2 and PKN3) forms. There is strong evidence that this family of kinases can be related to the PI3K/Akt/mTOR signalling pathway at some levels. As such, a deeper understanding could yield improvements not only in how their mutations and dysregulation play causal roles in human diseases, but may also provide insights to develop agonists and antagonists for use in therapy. In this respect, it is well known that PKC belongs to the AGC (cAMP-dependent, cGMP-dependent and protein kinase C) family which includes Akt [29]. PKC and Akt share some common kinases (i.e., PDK1 and mTORC2) that perform important regulatory functions [30–33]. Moreover, some PKC isoforms interact with Akt although their specific effect on Akt activity is isoform dependent [34, 35]; it has also been demonstrated that high glucose induced Akt activation, in a PKC*β*-dependent manner [36]. In addition, it has been found that activation of PKC*η* leads to the activation of Akt and mTOR signalling pathway, promoting glioblastoma cells proliferation [37]. PKC activity seems to be related also to PP2a and PTEN. PKC*δ* not only physically associates with the PP2a catalytic subunit (PP2a/C) but also phosphorylates the phosphatase to increase its activity [38]; this is the case for PKC*α* too, which seems to be the primary mediator of the ~2-fold increase in PP2a activity observed in intestinal cells after PKC-signalling activation [39]. Regarding PTEN, suppression of its activity by transforming growth factor-*β* (TGF-*β*) has been shown to be specifically mediated by PKC*α*. On the topic of PKCs, we will summarise their general characteristics and role in different cell signalling pathways, as well as the result of their deregulation in cell fate.

For reasons of brevity, we will not discuss much further the MAPK- and PKA-mediated pathways; interested readers should refer to [40–44].

## 2. Akt Kinase

The serine/threonine kinase Akt constitutes an important node in diverse signalling cascades downstream of growth factor receptor tyrosine kinases. Akt plays an essential role in cell survival, growth, migration, proliferation, polarity, metabolism (lipid and glucose), cell cycle progression, muscle and cardiomyocyte contractility, angiogenesis, and self-renewal of stem cells. Altered Akt activity has been associated with cancer and other disease conditions, such as diabetes mellitus, neurodegenerative diseases, and muscle hypotrophy [45].

Among the downstream effectors of PI3Ks, Akt is the most important and best studied [46]. Three Akt isoforms have been identified in mice and humans [47]. These three Akt proteins, although encoded by distinct genes localized on different chromosomes, have approximately 80% amino acid identity and similar domain structures. Each isoform possesses an N-terminal PH domain of approximately 100 amino acids, with a high similarity to PH domains found in other signalling molecules that bind 3-phosphoinositides [48]. Biochemical analysis revealed that the PH domain of Akt binds to both PIP3 and phosphatidylinositol 4,5-bisphosphate (PIP2) with similar affinity [49]. Moreover, in contrast to PH domains of other proteins, the head group of PIP3 is localized in a significantly different orientation in the PH domain of Akt, and it lacks a specific tyrosine that is conserved in PH domains of other proteins (e.g., DAPP1, GRP1, BTK) [50]. The kinase catalytic domain, located in the central region of the protein, shows a high degree of similarity to those found in PKA and PKC [51]: the relation with PKA and PKC explains the other name of Akt, PKB. Also present in this region is a threonine residue (T308 in Akt1) whose phosphorylation is necessary for the activation of Akt. Following the kinase domain is a C-terminal tail of around 40 amino acids, containing a second regulatory phosphorylation site (S473 in Akt1). This region possesses the F-X-X-F/Y-S/T-Y/F hydrophobic motif (where X is any amino acid) that is characteristic of the AGC kinase family. In mammalian Akt isoforms, this motif is identical (FPQFSY) [52]. Phosphorylation at T308 and S473 occurs in response to growth factors and other extracellular stimuli and is essential for maximal Akt activation [53].

The crystal structure of activated Akt was determined in 2002 [54] and underlines the fundamental role of the hydrophobic motif as an allosteric regulator of the kinase activity: in fact, with Akt phosphorylated on threonine in the catalytic loop but lacking its carboxyl-terminal hydrophobic motif, the *α*B and C-helices in the aminoterminal lobe of the kinase domain and the activation loop are disordered [55]. Replacing the hydrophobic motif with a similar sequence from the PKC-related kinase 2 (PRK2), containing an acidic residue instead of the Ser (called the PDK1-interacting fragment (PIF) sequence) [56], or mimicking the phosphorylation in S473 through a substitution with aspartate leads to a stabilization of the kinase domain in active state. In other words, the molecular interaction between the phosphorylated hydrophobic motif and the groove, formed by the *α*B and C-helices, stabilizes the whole catalytic domain: this association is critical for the complete activation of Akt.

As mentioned above, the activation of the PI3K signalling pathway by growth factor stimulation leads to recruitment of Akt to the plasma membrane through binding to PIP3. At the plasma membrane, Akt can be phosphorylated on two residues, T308 and S473, by two different kinases, PDK1 [22, 57] and the mTORC2 [23], respectively. Once activated, Akt translocates to various subcellular compartments, including the Golgi, ER, mitochondria, and nucleus [58], where it phosphorylates substrates or interacts with other molecules (Figures 1 and 3). Consensus motif analysis indicates that there are potentially thousands of cellular substrates for Akt; about 50 of these have been characterized so far. Through phosphorylation, Akt may either positively or negatively affect the function of these substrates, alter their subcellular localization, or modify their protein stabilities [45]. As a protooncoprotein and the primary target of PI3K, Akt was first characterized for its function in regulating cell survival and cell proliferation, and its antiapoptotic activity is solved through the inactivation of many proapoptotic factors. Constitutive activation of Akt leads to uncontrolled cell proliferation, inhibited apoptotic pathways, and strong cell cycle dysregulation, typical hallmarks of many human cancers. Akt is able to directly or indirectly modulate apoptosis [59]. The direct effects are linked to phosphorylation events or interactions with cell death actors, whereas the indirect regulation of apoptosis is mediated through transcriptional responses to apoptotic stimuli.

Active Akt migrates to both the cytosol and the nucleus. Nevertheless, the relative contribution of Akt signalling at the plasma membrane, the cytosol, and the nucleus remains to be elucidated. Nuclear Akt may fulfil important antiapoptotic roles [60] (Figure 1). Indeed, Akt plays a crucial role in determining cell fate by regulating fundamental transcriptional factors for the expression of pro- or anti-apoptotic molecules, for example, YAP, CREB, FOXO proteins, and NF-*κ*B, as well as the E3 ubiquitin ligase MDM2, a known negative regulator of the tumour suppressor p53 [61]. Phosphorylation of CREB and NF-*κ*B induce upregulation of antiapoptotic Bcl-2 family members, whereas phosphorylation of FOXO family members leads to their nuclear exclusion and inactivation [62, 63], with a consequent decreased transcriptional activity that is required for promoting apoptosis. Interestingly, Trotman et al. have shown that the promyelocytic leukemia protein (PML) tumour suppressor prevents cancer by inactivating phosphorylated Akt (pAkt) inside the nucleus. PML specifically recruits the Akt phosphatase PP2a as well as pAkt into PML nuclear bodies. PML-null cells are impaired in PP2a phosphatase activity towards Akt and thus, accumulate nuclear pAkt. As a consequence, the progressive reduction in PML dose leads to the inactivation of FOXO3a-mediated transcription of the proapoptotic Bim and the cell cycle inhibitor p27^kip1^ [64].

At the same time, Akt deliver antiapoptotic signals via different proteins directly modulated by Akt phosphorylation. Bad is one of the first discovered targets of Akt phosphorylation [65]. Bad is a proapoptotic member of the Bcl-2 family of proteins, able to bind Bcl-2 or Bcl-XL, blocking their antiapoptotic activities. Phosphorylation of Bad on S136 by Akt disrupts its interaction with Bcl-2/Bcl-XL, localized on the outer mitochondrial membrane, sequestering Bad in the cytosol, through the interaction with 14-3-3 protein. In an analogous way, phosphorylation by Akt of proapoptotic Bax protein on S184 suppresses its translocation to mitochondria, preventing Bax conformational change, a typical event that occurs after apoptotic induction [66]. In addition, the caspase cascade is further inhibited by Akt phosphorylation of procaspase 9 [67], inactivated through phosphorylation in S196, a residue that, however, is not conserved in other mammalian species [68]. Also Acinus, one of the most relevant proapoptotic factors, responsible for chromatin condensation, is phosphorylated by Akt in S422 and S573, with a consequent resistance of the protein to the activator cleavage by caspase 3 [69]. Moreover, Akt phosphorylates the two isoforms, *α* and *β*, of the glycogen synthase kinase 3 (GSK-3) in S21 and S9, respectively, promoting their inactivation [70] and blocking its ability to induce apoptosis in response to a wide range of stimuli (reviewed in [71]). Interestingly, GSK-3 *β* is a target of Akt phosphorylation/inactivation also inside mitochondria [72], but the role of this mitochondrial interaction remains to be clarified.

Akt is at the crossroads of several mitochondria-mediated cell death pathways and exerts a major role in apoptosis due to its networking with mitochondria in the regulation, and interconnection of metabolic pathways and cell survival. Notably, the mitochondrial import and export mechanisms for Akt have yet to be investigated. Also linked to mitochondrial metabolism is the Akt activity on hexokinase. Hexokinase catalyzes the first step of glycolysis: in particular, hexokinases I and II are found to directly interact with mitochondria, and their expression is increased in tumours [73], providing a putative explanation of the Warburg effect. In cancer cells, hypoxic conditions induce the activation of the PI3K/Akt cell survival pathway [74] and the association of Akt with mitochondria [75]. Akt promotes binding of hexokinase II to the mitochondrial voltage-dependent anion channel (VDAC) [76]. The high affinity of hexokinase II to VDAC allows it to selectively utilize intramitochondrial ATP to phosphorylate glucose and directly couple glycolysis to oxidative phosphorylation [77]. By promoting the interaction hexokinase-VDAC, Akt seems to maintain the “opening state” of the channel, protecting cells from apoptotic events through conservation of the mitochondrial integrity [76]. Despite this, other studies show how the hexokinase-VDAC binding mediates channel closure rather than VDAC opening, still maintaining the ability of hexokinases to inhibit cell death [78, 79], especially reducing the mitochondrial calcium (Ca^2+^) overload, a primary event that triggers cytochrome *c* release.

Connected to Ca^2+^-induced apoptosis, the inositol 1,4,5-trisphosphate receptor (IP3R), the main ER Ca^2+^-release channel, is phosphorylated by Akt due to the presence of a consensus substrate motif that is conserved in all the three IP3R isoforms. The consensus substrate motif (R-X-R-X-X-S/T) for Akt kinase is located in the C-terminal portion of the IP3R, and it is also conserved in IP3Rs cloned from several different species, with the exception of *Caenorhabditis elegans * [80]. The IP3R is involved in Ca^2+^ mobilization from intracellular stores, where channel activity is largely under the control of IP3 binding. This receptor is also involved in fundamental processes such as fertilization, mitosis, and apoptosis. In fact, there is general agreement in the literature that Ca^2+^ efflux from the ER and Ca^2+^ accumulation into mitochondria are linked to the effects of various apoptotic stimuli [81]. Akt, probably through its kinase activity, reduces Ca^2+^ release from the IP3R and protects the cells from apoptosis induced by several Ca^2+^-mediated apoptotic stimuli [82, 83]. Moreover, a recent paper by our group explains how the protein PML functions at the MAMs and ER levels to suppress Ca^2+^ transfer to the mitochondria. Knock-out cells for PML show a strong reduction of IP3R Ca^2+^ release, due to a hyperactivation of Akt. PML is a negative regulator of Akt, forming a macrocomplex composed by PML, Akt, the phosphatase PP2a, and IP3R type 3 at ER/MAMs. In the absence of PML, PP2a cannot inhibit Akt, with a consequent increase in IP3R phosphorylation, limited Ca^2+^ release, and protection from apoptosis [84] (see also below, PP2a section). Another protein regulated by Akt that mediates interorganelle signalling and transmits apoptotic signals from the ER to mitochondria is phosphofurin acidic cluster-sorting protein-2** (**PACS-2). Akt phosphorylates this protein in S473, favouring the binding with 14-3-3, which represses PACS-2 apoptotic activity [85].

One of the best-conserved functions of Akt is its role in promoting cell growth. The predominant mechanism appears to be through activation of the mammalian target of rapamycin complex 1 (mTORC1), which is regulated by both nutrients and growth factor signalling. Akt has been suggested to directly phosphorylate mTOR on S2448 [86], but the role of this phosphorylation remains still unclear. The mTORC1 activation by Akt seems due to the inhibitory phosphorylation of the tuberous sclerosis complex 2 (TSC2, also known as tuberin), a tumour suppressor, critical negative regulator of mTORC1 signalling [87]. This complex functions as a GTPase-activating protein (GAP) for the small G protein Ras homologue enriched in brain (RHEB). The decreased GAP activity of the complex leads to accumulation of RHEB-GTP and activation of mTORC1. However, it remains poorly understood how TSC2 phosphorylation by Akt leads to decreased GAP activity [88]. Moreover, a second Akt substrate has been found to be involved in mTORC1 regulation, the proline-rich Akt substrate of 40 kDa (PRAS40, also known as Akt1 substrate 1). Akt was shown to directly phosphorylate PRAS40 on T246 [89], and this phosphorylation was fundamental for 14-3-3 binding. PRAS40 associates with mTORC1, negatively regulates it; therefore, phosphorylation by Akt and association with 14-3-3 are crucial for insulin to stimulate mTOR [90]. Akt regulation of mTORC1 through TSC2 and PRAS40 phosphorylation is also important for cell proliferation, controlling the translation of proteins important for cell-cycle progression. Akt-mediated cell proliferation and oncogenic transformation has been shown to be dependent on mTORC1 activation [91], raising the possibility that mTORC1 is the dominant Akt target in cells transformed with mutant Ras [92].

Considering the strong connection between Akt and mTORC1 and the role of this signalling, frequently dysregulated in cancers, in the next section, we will focus our attention on the major component of the mTOR complex, the kinase mTOR.

## 3. mTOR Kinase

The PI3K pathway includes a number of critical effectors that are involved in basic cellular functions, such as cell growth control, the cell cycle and DNA damage checkpoints, and recombination or maintenance of telomere length. One of this family members is mTOR, now recognized as a central regulator in a diverse array of vital cellular processes, including proliferation, growth, differentiation, and survival [93]. The physiological importance of mTOR is undoubtedly demonstrated by the fact that the knockout of mTOR in mice is primordially embryonic lethal [94], and the dysregulation of the mTOR pathway is associated with increased transformation and oncogenesis [20].

Structurally, mTOR possesses in the N-terminus up to 20 tandem HEAT motifs, including a protein-protein interaction structure of two tandem antiparallel *α*-helices found in huntingtin, elongation factor 3, the A subunit of protein PP2a, and TOR [95]. The C-terminus consists of mutated FRAP-ataxia-telangiectasia (FAT), a transformation/transcription-domain-associated protein domain, an FRB domain (FKPB12 (FK506-binding protein 12 kDa)-rapamycin binding), a catalytic kinase domain containing also an ATP-binding site, a probable autoinhibitory or repressor domain, and an FATC (FAT carboxy-terminal) domain. The kinase domain is between the FRB domain (which is C-terminal to the FAT domain) and the FATC domain, located at the C-terminus of the protein [96]. It is speculated that the HEAT repeats serve to mediate protein-protein interactions, the FRB domain is responsible to provide a docking site for the FKBP12/rapamycin complex, and FAT and FATC domains modulate mTOR kinase activity via unknown mechanisms [97]. The catalytic kinase domain in the C-terminus has a high similarity to the catalytic domain of PI3K, so mTOR is considered a member of the PIKK (PI3K-related kinase) family, but there is no experimental evidence that it displays lipid kinase activity [98]. In mammalian cells, mTOR exists in two distinct complexes called complex 1 (mTORC1) and complex 2 (mTORC2).

mTORC1 is rapamycin-sensitive and consists of the mTOR catalytic subunit, Raptor (regulatory associated protein of mTOR), mLST8 (also known as G*β*L), and PRAS40 [99]. Whereas the function of mLST8 is not really clarified, Raptor regulates mTORC1 functioning as a scaffold for recruiting mTORC1 substrates. PRAS40 is phosphorylated by Akt at T246 releasing its inhibitory effects on mTORC1. mTORC1 is a master controller of protein synthesis, integrating signals from growth factors within the context of the energy, and nutritional conditions of the cell. Activated mTORC1 regulates protein synthesis by directly phosphorylating 4E-BP1 (eukaryotic initiation factor 4E binding protein-1) and p70S6K (ribosomal p70S6 kinase), translation initiation factors that are important to cap-dependent mRNA translation, which increases the level of many proteins that are needed for cell cycle progression, proliferation, angiogenesis, and survival pathways [100]. Many diverse signals (such as growth factors, amino acids, glucose, energy status, and different forms of stress) and pharmacological agents (such as rapamycin) regulate the mTORC1 pathway.

In the other complex, mTORC2, mTOR also contains the mLST8 protein and additionally interacts with a protein called Sin1 (stress-activated protein kinase-interacting protein), a second protein termed Protor (protein observed with Rictor), and, instead of Raptor, Rictor (rapamycin-insensitive companion of mTOR) is present [101] (Figure 1). The interaction between Rictor and mTOR is not blocked by the drug rapamycin nor affected by nutrient levels, which are conditions known to regulate mTORC1. mTORC2 modulates cell survival in response to growth factors by phosphorylating Akt on Ser473 which enhances subsequent Akt phosphorylation on Thr308 by PDK1 [23]. Another downstream target of mTORC2 is serum/glucocorticoid-regulated kinase 1 (SGK1) [102]. Also the hydrophobic motif phosphorylation of PKC*α* has been shown to be mediated by mTORC2 [103]. Moreover, mTORC2-mediated phosphorylation of Akt and conventional PKC (cPKC) in their C-terminal turn motif (TM) is crucial for their proper carboxyl-terminal folding, stability, and signalling [32, 33].

The mTOR signalling pathway is activated during a wide variety of cellular responses: it regulates growth by maintaining the appropriate balance between anabolic processes, such as macromolecular synthesis and nutrient storage, and catabolic processes, like autophagy and the utilization of energy stores. Active mTOR enhances cell growth promoting protein translation and increasing cell mass by controlling a subset of mRNAs that are thought to promote cell growth and proliferation.

Most of protein translation is modulated at the level of initiation, through the positioning of the ribosome at the AUG codon. Cellular mRNAs contain a cap structure at their 5′ terminus [104], and it has been demonstrated that a strong repressor of cap-mediated translation is 4E-BP1 [105]. mTOR phosphorylates and inactivate the translation inhibitor 4E-BP1, inducing its dissociation from the translation initiation factor eIF4E, which can bind the cap structure at the 5′ termini of mRNAs, thereby allowing cap-dependent translation (in fact, dephosphorylated 4E-BP1 binds and inhibits eIF4E) of proteins involved in cell proliferation and survival [106]. Moreover, mTOR is able to activate the p70S6K (through T389 phosphorylation) which activates the 40S ribosomal protein S6 via phosphorylation at S240/244 [107], a required process for translation and cell growth. mTOR is also able to monitor the regulation of ribosome biogenesis, an aspect that fundamentally occurs during the translation of mRNAs of the ribosomal proteins and the synthesis of ribosomal RNA (rRNA). Transcription of ribosomal DNA (rDNA) and transfer RNA (tRNA) genes by RNA polymerase I (Pol I) and III (Pol III) is a major rate-limiting step in the biogenesis of ribosomes. Nuclear localized mTOR is involved in Pol-I- and Pol-III-mediated transcription of rDNA and tRNA genes. It has been demonstrated that mTOR is associated with the promoters of 45S rDNA and genes of 5S rDNA and tRNAs [108]. mTOR also regulates Pol-I and Pol-III-mediated transcription through phosphorylation of TIF-IA and upstream binding factor (UBF), two transcription initiation factors of Pol I [109, 110]. The phosphorylation of TIF-IA in Ser44 is indispensable for its activity, while phosphorylation in Ser199 inhibits TIF-IA. Indeed, it has been demonstrated that inhibition of mTOR signalling inactivates TIF-IA by decreasing phosphorylation at Ser44 and enhancing phosphorylation at Ser199 [111]. Additionally, this effect not only regulates the activity of TIF-IA, but also controls its intracellular localization. Treatment of cells with mTOR inhibitors causes translocation of a significant part of TIF-IA into the cytosol (from the nucleus) inhibiting the formation of the transcription-initiation complex [111].

The signalling components upstream and downstream of mTOR are frequently altered in a wide variety of human tumours. Mutations in several tumour-suppressor genes (such as TSC1, TSC2, LKB1, *PTEN*, *VHL*, NF1, and PKD1) trigger the development of different diseases [100]. To confirm a primary role of mTOR in cancer development, it was shown that the inhibition of this kinase is fundamental for blocking cell growth and motility in a number of tumour cell lines [112]. Different data provide evidence that the mTOR pathways receive stimulatory signals from Ras, and, ultimately, these pathways drive tumorigenesis through the coordinated phosphorylation of proteins that directly regulate protein synthesis, cell-cycle progression, and cancer development. It has been demonstrated that Ras is able to determine an ERK-dependent phosphorylation of TSC2. This results in the suppression of its biochemical and biological tumour-suppressive functions (the loss of TSC2 is the typical aspect of the tumour syndrome tuberous sclerosis) and in the activation of S6K through the mTOR-dependent site T389. This shows a direct control of translation through a mechanism that involves the TSC1/2-mTOR pathway and Ras-MAPK (mitogen-activated protein kinase) signalling [113].

mTOR is also able to regulate the expression of the hypoxia-inducible factor-1 alpha (HIF-1*α*). Hypoxia occurs in the majority of tumours, promoting angiogenesis, metastasis, and resistance to therapy. Responses to hypoxia are orchestrated in part through the activation of the hypoxia-inducible factor family of transcription factors (HIFs) [114]. HIFs are heterodimeric transcription factors composed of an *α* and a *β* subunit (whose levels are controlled by oxygen tension [115]) and activates the transcription of 100–200 genes involved in cellular metabolism and the adaptation of cells to hypoxic conditions. A number of interesting potential connections have emerged between HIFs and mTOR, and there are clear examples of these pathways regulating each other and common downstream pathways. Perhaps the most firmly established is the ability of mTOR to influence HIF1A translation: mTORC1 signalling increases HIF-1*α* protein levels by promoting mRNA translation from its 5′UTR [116–118]. At the same time, hypoxia and low oxygen levels inhibit mTOR signalling through multiple mechanisms. One of these determines a decrease in cellular ATP levels with consequent activation of AMPK (AMP-activated protein kinase), which inhibits mTOR via TSC2 and raptor phosphorylation [119].

Moreover, the mTOR pathway is the most studied pathway regulating mammalian autophagy. Autophagy, which is highly conserved from yeast to humans, is a bulk degradation process involved in the clearance of long-lived proteins and organelles. Dysfunction in the autophagy pathway has been implicated in an increasing number of human diseases, from infectious diseases to cancer and neurodegeneration. mTOR negatively regulates autophagy, and, downstream of this kinase, there are more than 30 different genes regulating autophagy in yeast (known as the *ATG *genes), and many of these have mammalian orthologs [120]. mTORC1 regulates numerous proteins that are required for the execution of the autophagic program, including a macromolecular complex (FIP200,ULK1/Atg1) implicated in the initiation step of autophagosome formation. mTORC1 is able to phosphorylate ULK1 (unc-51-like kinase 1), moderately reducing ULK1 kinase activity and, consequently, blocking autophagy [121] (Figure 1).

Despite the central role of mTOR in cell physiology, relatively little is known about its precise subcellular distribution and the underlying functional significance. Essentially, mTOR is localized in the cytosol where it plays the important function of maintaining an appropriate balance between anabolic and catabolic processes. However, following different stimuli, mTOR can be driven into other compartments where it performs its activity (Figure 3). Previous studies indicate that mTOR is present in both the cytoplasm [122] and nucleus [123, 124]. Biochemical characterization in association with confocal microscopy identified a new localization of mTOR in the ER and the Golgi apparatus; specifically, mTOR is a peripheral ER membrane protein, tightly anchored to the ER/Golgi membranes [125]. ER and Golgi localization sequences of mTOR have also been identified [126], suggesting that anchoring to the ER/Golgi is important for mTOR signal transduction. ER and the Golgi apparatus are internal membrane structures constituting more than one-half of the total membranes in a cell. These compartments have an important role in processing and packaging macromolecules and sequestration of Ca^2+^, which has been involved in mechanisms capable of activating both cell suicide programs as well as prosurvival mechanisms [127]. It is, therefore, possible to hypothesize that mTOR could play its role of amino acid sensor in the ER/Golgi side. Moreover, the Rag GTPase family has been shown to be an amino acid-specific regulator of mTOR [128], a condition that promotes the translocation of the kinase to a surface of endomembrane compartments, identified recently as the lysosomal surface. In fact, a trimeric complex, named Ragulator, (encoded by the genes *MAPKSP1*, ROBLD3, and c11orf59) interacts with the Rag GTPase, localizing these and mTOR to lysosomes, a translocation necessary for mTOR activity [129].

Recent data suggest that mTOR signalling might also regulate mitochondrial function [130]. Cunningham et al. have shown that mTOR is necessary for the maintenance of mitochondrial oxidative function. mTOR inhibition determines lower oxygen consumption, mitochondrial membrane potential, and cellular ATP levels. Conversely, hyperactivation of mTORC1 increases mitochondrial DNA copy number, as well as the expression of many genes encoding proteins involved in oxidative metabolism [131]. Interestingly, starting from the observation that mTOR regulates cell growth by sensing nutrients, ATP levels, and osmotic stress, and since energy metabolism is a critical aspect of mitochondria, Desai and coworkers demonstrated a direct interaction of a large portion of mTOR with mitochondria, exactly at the level of the outer mitochondrial membrane. As validation of this direct control of mitochondrial function by mTOR, induced mitochondrial dysfunction results in mTOR-mediated growth regulation, suggesting the presence of a critical crosstalk between mitochondrial activity and growth regulation mediated by mTOR [122]. Even if the mechanism remains still unclear, mTOR might be localized to mitochondria via FKBP38 (a member of the FK506-binding family of proteins), a mitochondrial protein that should bind to the FRB domain of mTOR [132]. Moreover, a recent study has identified mTOR in a complex with the mitochondrial outermembrane proteins Bcl-XL and VDAC1 and demonstrated that Bcl-XL, but not VDAC1, is a kinase substrate for mTOR in vitro; the employment of mTOR inhibitors caused the dissociation of mTOR from Bcl-XL, modulating the mitochondria metabolism [133].

As reported above, mTOR is strongly involved in growth, survival, metabolism, and cancer development. A variety of transformed or tumour cells with deregulated mTOR signalling have shown higher susceptibility to inhibitors of mTOR than normal cells. The identification of mTOR as a potential target for anticancer therapeutics occurred after the discovery of the antineoplastic properties of the natural product rapamycin. This lipophilic macrolide was isolated from a strain of *Streptomyces hygroscopicus *indigenous to Easter Island (known as Rapa Nui) more than 20 years ago [134]. Rapamycin was initially developed as an antifungal agent. However, its major application quickly changed after rapamycin was proven to have immunosuppressive and antiproliferative properties. Upon entering the cells, rapamycin binds the small protein receptor called FKBP12. The rapamycin/FKBP12 complex specifically binds to mTOR and potently interferes with its function, inhibiting signals required for cell cycle progression, cell growth, and proliferation, leading to cell-cycle arrest in the G1 [97]. This inhibition blocks the activation of two fundamental downstream signalling effectors: p70S6K and 4E-BP1. By inhibiting cell cycle progression, growth, and division, rapamycin (and its analogues, termed rapalogs) produce several immunosuppressive and antiproliferative effects. These molecules were expected to become a breakthrough for the treatment of different types of cancer. However, rapamycin and rapalogs demonstrated only a limited clinical efficacy. This is explained since the complex rapamycin/FKBP12 is able to inhibit mTORC1 only partially, and in addition mTORC2 is resistant to rapamycin. Moreover, there is another possible outcome: feedback inhibition of PI3K signalling may be relieved by rapamycin treatment, presumably by inhibiting the downstream activation of S6K, which can serve as a negative regulator of PI3K signalling [135]. In this situation, inhibition of mTORC1 relieves a feedback inhibition of PI3K and results in enhanced PI3K signalling to Akt. To bypass these issues, a new molecule directed at both mTOR complexes simultaneously was reported recently. This inhibitor binds to the ATP-binding site in the mTOR catalytic domain, inhibiting mTOR completely (both mTORC-1 and -2) and minimizing the feedback activation of PI3K-Akt signalling [136].

Despite its humble beginning two decades ago, the protein kinase mTOR is now recognized as a critical mediator of the cellular response to many types of stress, including DNA damage as well as drops in the levels of energy, glucose, amino acids, and oxygen. The physiological functions of mTOR continue to expand, and it has been demonstrated that mTOR is involved in a wide variety of human diseases. Recent data suggest that use of mTOR inhibitors result in antitumor activity against several types of refractory malignancies. It is hoped that a more detailed understanding of the mTOR signalling cascade will lead to new therapies for many different human disorders.

## 4. PP2a Phosphatases

PP2a is a family of serine-threonine phosphatases highly conserved and ubiquitously expressed, implicated in the control of diverse cellular processes through the negative regulation of signalling pathways initiated by protein kinases. PP2a phosphatase activity has been linked to the regulation of the cell cycle, signal transduction, DNA replication, transcription, and translation [137, 138].

The structure of PP2a has been extensively studied. PP2a is a heterotrimer consisting of a catalytic subunit (the 36 kDa C subunit, PP2a/C), a structural subunit (the 65 kDa A subunit, PP2a/A), and a regulatory subunit (the B subunit, PP2a/B, which can vary in size from 50 to 130 kDa). The A and C subunits are ubiquitously expressed and form a catalytic complex (PP2a/AC) that interacts with at least three families of regulatory subunits (B55, B56, and PR72/130) and tumour antigens (e.g., SV40 small T antigen). The generation of a great diversity of PP2a holoenzymes is due to the existence of several isoforms for each subunit and their combination. Numerous studies implicate specific roles for PP2a subunits in regulating physiological functions. The variable B-type subunits are expressed differentially by tissue and temporally during differentiation and/or development. They exert a crucial role in the control of PP2a activity by regulating substrate selectivity and regulating the catalytic activity in a wide range of biological processes [139]. In addition, there is evidence that the regulatory B subunits may target the catalytic complex to intracellular sites such as microtubules, the nucleus, and the mitochondria (Figures 1 and 3) [140, 141]. Some PP2a hetero-trimers display a quite restricted subcellular distribution [142]. Nuclear localization signals have been identified experimentally in B56*α*, and B56*ε*, while nuclear export signals are present in B56*α*, B56*β* and B56*ε* [143, 144]: as a consequence, B56 proteins can be detected at centromeres (B56*α* colocalized with pericentrin, a centrosomal marker) from prophase to metaphase during mitosis [145], while during interphase, B56*α*, B56*β*, and B56*ε* undergo nuclear-cytoplasmic shuttling, with a prevalent localization in the cytoplasm [139]. Nuclear PP2a proteins containing different B56 isoforms exert a further function in maintaining the stability of cohesin at centromeres. The accurate chromosome segregation during mitosis and meiosis depends on shugoshin proteins that associate with PP2a/B56, which is thought to dephosphorylate cohesin and thereby, protecting cohesin degradation and prophase chromosome dissociation [146]. Although most regulatory subunits do not contain obvious targeting motifs, their presence certainly influences the compartmentalization of PP2a, such as focal adhesions [147] and the microtubule cytoskeleton [142]. Moreover, ceramide, the central molecule of sphingolipid metabolism which generally mediates antiproliferative responses, such as cell growth inhibition, apoptosis induction, senescence modulation, ER stress responses, and/or autophagy, was found to promote the upregulation of a regulatory subunit (B56*α*) and thus promotes translocation of PP2a to the mitochondria. C2-ceramide, but not inactive C2-dihydroceramide, was found to specifically activate a mitochondrial PP2a, which rapidly and completely induced Bcl-2 dephosphorylation and correlated closely with ceramide-induced cell death [148, 149].

This explains partially the variety of specific physiological functions carried out by the enzyme and underlines the potential complexity of PP2a's role and its regulation [150] in several signalling pathways by regulating the activity of protein kinases, protein phosphatases, or their substrates [151]. Hence PP2a activity needs to be tightly regulated, and this is achievable through enzyme modulation at different levels. Therefore, in addition to the heterooligomeric composition of the PP2a complexes, which is the most important one, PP2a can also undergo posttranslational modifications and be controlled by its association with inhibitory proteins [150]. Specifically, the highly conserved C-terminal sequence of the PP2a catalytic subunit contains tyrosine residues, including Y307, which can be phosphorylated, resulting in inactivation of the enzyme [152], and PP2a can reactivate itself by autodephosphorylation. At least two endogenous protein inhibitors, designated I1PP2a (also known as pp32 or PHAP-1) and I2PP2a (also known as SET), were identified as noncompetitive inhibitors of PP2a activity [153]. The biological significance of I1PP2a is still mostly unknown. Conversely, I2PP2a is known to be phosphorylated on two serine residues (most likely by PKC) and is required in chromatin remodelling. The importance of its potent inhibitory activity towards PP2a is underlined in its involvement in a form of acute non-lymphocytic myeloid leukemia with t(6,9) and in BCR/ABL-driven leukemias (CML and Ph1-ALL), suggesting that disruption of normal PP2a regulation may also play a role in the pathogenesis of these leukemias [150]. Moreover, protein-protein interactions between PP2a and other intracellular components contribute to the specificity of PP2a signalling: the selective association of PP2a with scaffolding proteins directs the phosphatase to specific signalling modules [154]. 

Interaction of PP2a with other proteins might also recruit PP2a activity towards specific substrates in apoptotic signalling. PP2a promotes cell survival by negatively regulating the PI3K/Akt pathway, by associating to and directly dephosphorylating Akt, with consequent inactivation of the kinase [155] (Figure 1). Trotman et al. have shown that Akt and PP2a are corecruited into PML nuclear bodies and that the inability to recruit PP2a to Akt in *PML* deficiency resulted in an accumulation of nuclear phosphorylated Akt, which drives tumorigenesis [64]. Moreover, PML, Akt, and PP2a colocalize with the IP3R3 in high molecular weight complexes at the ER and MAMs, supporting a model in which dephosphorylation of Akt at the MAMs might occur through PML-mediated recruitment of PP2a. Reduced cellular sensitivity to apoptotic stimuli was observed in cells with high Akt activity, as a result of diminished Ca^2+^ flux from the ER through the IP3R. This effect is highly specific to Ca^2+^-mediated apoptotic stimuli and explains a more direct role of PP2a in regulating cell survival through changes in Ca^2+^ signalling in the ER, cytosol, and mitochondria [84]. Also in the sarcoplasmic reticulum of cardiac muscle cells, PP2a has been shown to generate a macromolecular complex with the ryanodine receptor Ca^2+^-release channel, the FK506-binding protein FKBP12.6, PKA, PP-1, and the muscle A-kinase anchoring protein (AKAP), where it is probably involved in the regulation of channel activity [156, 157].

The PP2a proapoptotic function is also mediated by direct dephosphorylation of specific proteins: PP2a both inactivates the antiapoptotic Bcl-2 and activates the proapoptotic factor Bad (Figure 1). Dependent on the survival stimuli and cell type, Bad becomes phosphorylated at S112, S136 and/or S155 by different prosurvival kinases such as mitochondrial cAMP-dependent protein kinase (PKA) and cytosolic Akt, which cooperatively mediate binding of Bad to 14-3-3 proteins, causing cytoplasmic retention of Bad. This interferes with the ability of Bad to translocate to the mitochondrial membrane and to inhibit the antiapoptotic Bcl-2 protein. In the absence of survival stimuli, Bad is dephosphorylated and colocalises and binds with prosurvival Bcl-2 members at the mitochondrial membrane, leading to apoptotic cell death. Finally, the A/PR65 subunit of PP2a is a substrate of caspase-3. Hence, partial or total loss (by further degradation) of the A/PR65 subunit resulted in an increase in PP2a activity towards yet unidentified substrates, which leads to the increase of the apoptotic response [138].

A direct involvement of PP2a in the regulation of the cell cycle has been extensively demonstrated. The distribution of the protein differs with cell-cycle phase, is markedly accumulated in the nucleus at the G1/S border and in S phase, regulating the G1- to S-transition [158]. PP2a activity is also modulated by cell cycle-related events: indeed, the methylation levels of nuclear and cytoplasmic PP2a vary during the G0/G1 and G1/S phases [154]. PP2a is required in the control of the Cdk1/cyclin B complex (also known as maturation-promoting factor, MPF) [142]. In early G2, PP2a, containing a B56*δ*/B55*α*-targeting subunit [159], keeps MPF in the inactive precursor state by inhibiting the activities of both CAK (Cdk-activating kinase) and Wee1 kinase [156, 160]. Together, CAK, Wee1, and the dual-specificity kinase Myt1 switch off Cdk1/cyclin B [142]. PP2a also inhibits complete phosphorylation of Cdc25 [156], which is a critical upstream regulator of Cdk1 [160]. Indeed, final activation of MPF occurs when the inhibitory Thr14 and Tyr15 are dephosphorylated by the dual-specificity phosphatase Cdc25. PP2a is also positively implicated in the exit from mitosis, mediating cyclin B destruction [156]. Lastly, full mitotic exit requires the inhibition of both PP2a/B55*α* and PP2a/B55*δ* complexes, which is obtained by the action of the kinase Mast-I (Greatwall), a target of Cdk1/cyclin B. Once this system is active, the cell switches to complete mitosis, implying that inhibition of protein phosphatases is critically important for this process [156, 159, 161].

Moreover, PP2a can dephosphorylate and inactivate MEK1 and ERK-family kinases, therefore, inhibiting mitogenic signals; this effect is obtained directly and indirectly, through the inactivation of PKC*ζ* enzyme [162], which in turn stimulates the MAPK cascade through Raf-1 independent activation of MEK [150, 154]. Accordingly, PP2a exerts its anti-mitogenic function through the negative regulation of the eukaryotic initiation factor 4E (eIF4E) and eIF4F assembly, which requires the MAPK Mnk1/2 signalling mediating eIF4E phosphorylation at S209. Inhibition of PP2a causes increased Mnks and eIF4E phosphorylation, resulting in the dissociation of eIF4E from 4E-BP1 and the subsequent increase in eIF4F assembly, potentiating the expression of certain oncogenic proteins such as Mcl-1 and c-Myc [163] (Figure 1). The SV40 small t-antigen too, a well-known PP2a inhibitor and a cell-transforming viral protein, is believed to transform cells in part by hyperactivation of the MAPK pathway as a consequence of PP2a inactivation [150].

PP2a can also dephosphorylate MDM2 at T216, and this is thought to activate MDM2 resulting in the degradation of p53, a proapoptotic transcription factor [164]. PP2a inhibits the mTOR pathway too, through direct inactivation of the S6 kinase, which is responsible for promoting cell growth [150]. Other PP2a substrates involved in prosurvival signals are proteins integrated in the signal-dependent activation of the NF-*κ*B pathway, which indeed regulates the expression of multiple genes involved in the control of cell growth, division, and survival. Specifically, PP2a dephosphorylates the NF-*κ*B protein family members RelA [150]. PP2a interacts with IKK in the multicomponent protein kinase termed the IKK signalosome, facilitating the induction of IkB kinase activity, targeted degradation of IkB, and release of NF-*κ*B to its nuclear site of action following the stimulation with the proinflammatory cytokine TNF. The precise mechanism of PP2a action on IKK remains unclear. However, given the key role that IKK plays in inflammation, the kinase/phosphatase interaction described represents an attractive therapeutic target for small molecule inhibitors [165].

By altering the functions of proteins involved in mitogenic and apoptotic signalling pathways, PP2a is considered to be a tumour suppressor, and many observations support a role for PP2a in tumorigenesis. Indeed, the dysfunction of particular PP2a complexes regulates specific phosphorylation events necessary for cancer initiation. Moreover, the disruption of functional PP2a complexes also induces transformation. Mutations of both structural subunits contribute with low frequency to different subsets of human cancers by distinct mechanisms [166]. In conclusion, characterization of the specific physiological substrates and the regulation of PP2a holoenzyme assembly will be one of the main requirements to identify the in vivo function and regulation of PP2a in different signal transduction pathways.

## 5. PTEN Phosphatase

In 1997, two groups independently searching for tumour suppressors on chromosome 10q23, a locus that is frequently deleted in a variety of human cancers [167], identified a new phosphatase termed PTEN (phosphatase and tensin homolog deleted on chromosome 10), also known as MMAC (mutated in multiple advanced cancers) [168] or TEP1 (transforming growth factor-*β*-regulated and epithelial-cell enriched phosphatase 1) [169]. Subsequently, *PTEN* somatic mutations were identified in almost all human tumour types, especially those of the brain (above all glioblastoma), prostate, and endometrium; moreover, mutation of at least one allele occurs in one-third or more of breast, colon, and lung tumours [170]. Therefore, nowadays the PTEN gene appears to be the second most frequently mutated tumour-suppressor gene in human cancers after TP53 [171]. Germline mutations in PTEN are associated with a wide and diverse clinical spectrum of inherited autosomal dominant disorders characterized by developmental disorders, neurological deficits, multiple hamartomas, and an increased risk of breast, thyroid, and endometrial cancers. Collectively, these are referred to as the PTEN hamartoma tumour syndromes (PHTS), which include Cowden syndrome (CS), Lhermitte-Duclos disease, Bannayan-Riley-Ruvalcaba syndrome, and Proteus and Proteus-like syndromes [172]. Some of the patients with these disorders also show macrocephaly, mental retardation, ataxia, and seizures. Recently, a subset of autistic patients with macrocephaly were found to bear PTEN mutations [173]. Various mouse models in which the *PTEN *gene is deleted further demonstrate its crucial role as a tumour suppressor in multiple tumour types, and the consequences of its loss in human health and disease (for recent reviews, see [174, 175]). Notably, these studies revealed the requirement of PTEN for embryonic development and provide evidence for its haploinsufficient tumour-suppressive activity (one functional allele is not enough to sustain a wild-type condition) [176–180]. Today, it is well documented that PTEN function affects diverse cellular processes such as cell-cycle progression, cell proliferation, apoptosis, ageing, DNA damage response, angiogenesis, muscle contractility, chemotaxis, cell polarity, and stem cell maintenance.

As its name suggests, PTEN has been shown to be a non-redundant, evolutionarily conserved dual-specificity phosphatase that is capable of removing phosphates from protein and lipid substrates [181, 182]. PTEN harbours the Cys-X_5_-Arg-Thr/Ser (CX_5_RT/S) phosphatase catalytic signature and shares primary sequence similarity with members of the protein tyrosine phosphatase superfamily [183]. This initially suggested that PTEN is a protein phosphatase and prompted the expectation that its function would be to suppress directly oncogenic tyrosine kinase signalling [184]. Indeed, early studies showed PTEN-dephosphorylated serine, threonine, and tyrosine residues in peptide substrates in vitro [185] and focal adhesion kinase (FAK) in vivo [186]. Studies carried out so far have firmly established that the protein phosphatase activity of PTEN plays a role in the regulation of cellular processes, especially cell migration [187, 188]. In addition, there are reports that PTEN can dephosphorylate itself [189], the platelet-derived growth factor receptor [190], and various studies have demonstrated that the protein phosphatase activity of PTEN regulates MAPK phosphorylation and cyclin D1 expression [191, 192].

Ironically, although PTEN represents the only protein tyrosine phosphatase that can unambiguously be termed a tumour suppressor, it is its lipid phosphatase function that has been shown to be crucial for maintaining tissue homeostasis [181]. Specifically, the primary target of PTEN in cancer is the lipid second messenger intermediate PIP3 [182]. PTEN removes the phosphate from the three-position of the inositol ring to generate PIP2, thereby, directly antagonizing signalling through the PI3K/Akt pathway [8, 26, 193, 194]. The loss of *PTEN *leads to constitutively high levels of PIP3, which promotes the recruitment of a subset of proteins that contain a pleckstrin homology domain to cellular membranes, including PDK1 and Akt kinase. As discussed before, Akt is the major downstream effector of PI3K signalling that can phosphorylate a wide array of substrates and, thus, stimulates cell growth, proliferation, and survival (Figure 1) [45].

The obvious biological effect caused by a modest change of PTEN expression level in mouse models also emphasizes that PTEN function needs to be precisely controlled in a temporally and spatially specific manner. Yet, the function and regulation of PTEN has turned out to be amazingly complex. PTEN can be regulated through multiple mechanisms as well as epigenetic effects: transcriptional modulation, posttranscriptional mechanisms (also through coding-independent function of pseudogene PTENP1 mRNA, as recently discovered [195]), posttranslational modifications (including oxidation, acetylation, phosphorylation, and ubiquitination), binding partners, and subcellular localization—all these potentially impact PTEN levels and/or function. Every one of these mechanisms has been extensively reviewed elsewhere [196–198]; here, we will only provide a brief overview, with particular emphasis on new perspectives that have emerged about the importance of PTEN's subcellular localization (Figures 1 and 3).

Human PTEN encompasses 403 amino acids and is characterized by five functional domains: a short N-terminal PIP2-binding domain, a phosphatase domain, a C2 domain, a C-terminal tail containing PEST (proline, glutamic acid, serine, threonine) sequences, and a PDZ (postsynaptic density protein—*Drosophila* disc large tumour suppressor—zonula occludens 1 protein) interaction motif [26]. The majority of the missense mutations found in human tumours and CS occur in the phosphatase domain and affect the catalytic activity of PTEN [199]. Nevertheless, mutations also occur in all other domains of PTEN, strongly suggesting that these different domains are physiologically relevant to PTEN-related tumorigenesis.

PTEN's amino acid sequence and tertiary structure explain a great deal about PTEN's unique enzymatic properties. Detailed analysis of the PTEN crystal structure [200] revealed that its phosphatase domain contains all the highly conserved residues of the signature motif CX_5_RT/S present in the active sites of protein tyrosine phosphatases and dual-specificity phosphatases, required for protein phosphatase action but, in addition, has two unique Lys residues that might interact with negatively charged PIPs. Moreover, the PTEN active site pocket is wider and slightly deeper than other protein phosphatases, allowing both bulky PIP3 and smaller phosphoaminoacids to be substrates. However, the shape of the active site pocket, electrostatic interactions with positively charged sidechains, and hydrogen bonds with polar sidechains make PIP3 binding more favourable [201]. Interestingly, PTEN protein- and phosphoinositide-phosphatase activities can be uncoupled. For example, the C124S mutation (which is the catalytic site residue) inactivates both lipid and protein phosphatase activity [181] but the G129E mutation disrupts the lipid but not the protein phosphatase activity of PTEN [182]. The presence of this mutation in patients with PHTS and sporadic human tumours clearly indicates that loss of the lipid phosphatase activity is sufficient to cause these clinical phenotypes. The unimpressive activity of PTEN towards phosphopeptide substrates, while consistent with the idea that it is primarily a lipid phosphatase, does not exclude the possibility that PTEN is also a protein phosphatase, but one with an exquisite substrate specificity, as recently demonstrated [183].

Even though PTEN has multiple domains for membrane association, in most mammalian cell types it does not show an obvious association with the plasma membrane. Instead, PTEN usually appears diffusely in the cytosol, with a somewhat variable nuclear component. The crystal structure revealed that PTEN has a C2 domain that harbours basic residues essential for phospholipid membrane binding in vitro [202]. In addition, the C-terminal, apparently unstructured and flexible tail of PTEN, contains a cluster of serine and threonine phosphorylation sites (S370, S380, T382, T383, and S385) which may regulate its stability, activity, and recruitment to the membrane [203]. Multiple kinases including casein kinase 2 (CK2) [204], GSK3*β* [205], PICT-1 [206], and Rock [207] are capable of phosphorylating the PTEN C-terminal tail. Vazquez et al. have proposed that when phosphorylated PTEN assumes a closed conformation (due to interaction between the phosphorylation cluster of the C-terminal tail and the positively charged surface on the C2 domain), this enhances its stability but opposes membrane binding, thus keeping PTEN inactive in the cytoplasm (in terms of its lipid phosphatase activity) [208, 209]. Dephosphorylation leads to a more active phosphatase, but also more unstable and subject to ubiquitin-mediated proteasomal degradation (Figure 1). The identity of PTEN's phosphatase is still unclear (and also how it might be activated), but it has been proposed that PTEN could be its own phosphatase, dephosphorylating itself [189].

More recently, infrared spectroscopy experiments have also indicated that the PTEN N-terminal domain can bind specifically to PIP2; this is involved in PTEN membrane targeting and, in addition, causes a conformational change within the enzyme and its allosteric activation [210, 211]. Single-molecule studies using total internal reflection fluorescence microscopy (TIRF) have shown that PTEN associates stably but transiently (about 150 ms) with the plasma membrane [212], and localization of PTEN to the plasma membrane is presumably influenced by the local PIP2 and PIP3 concentrations [213]. As such, PTEN is an “interfacial enzyme”, which exists in a high-activity state when bound transiently at membrane surfaces containing its substrate and other acidic lipids, such as PIP3 and phosphatidylserine. This mechanism ensures that PTEN functions in a spatially restricted manner and may explain its involvement in forming the gradients of PIP3, which are necessary for generating and/or sustaining cell polarity during motility, in developing neurons and in epithelial tissues [214]. Moreover, other potential functional consequences of this are suggested by experiments that identified pools of PIP3 in the nuclear matrix and on the ER and other endomembrane compartments [215, 216]. 

The identification of nuclear PIP2 and PIP3 [217, 218], as well as other key components of the PI3K pathway including PI3Ks, PDK1, and Akt, indicate that PTEN might also function as a PIP3 lipid phosphatase in the nucleus [219]. It is now well documented that a pool of PTEN protein is located and functional within the nucleus; accumulating evidence also suggests that the role of nuclear PTEN is not the same as that of cytoplasmic PTEN. Importantly, loss of this nuclear PTEN population correlates with increased tumorigenicity [220–222]. PTEN nuclear shuttling is mediated by ubiquitination; as described above, PTEN contains the two so-called PEST motifs characteristic of short-lived proteins that are subject to ubiquitin-mediated degradation by the proteasome. In support of this, Wang et al. [223] have identified NEDD4-1 as an E3-ligase for PTEN. In addition to promoting polyubiquitination and, therefore degradation of PTEN, NEDD4-1 can also catalyse monoubiquitination of PTEN, which was shown by Pandolfi's laboratory to promote PTEN nuclear import (Figure 1) [224]. The same group have also identified HAUSP as a critical and essential enzyme for PTEN deubiquitination and nuclear exclusion, a mechanism which involved also the tumour-suppressor PML [225]. Nuclear PTEN affects a variety of biological functions: chromosomal stability through an interaction with the centromeric-binding protein CENP-C, which enhances centromere stability specifically and overall genomic stability [226]; DNA-damage responses through its ability to upregulate the transcription of Rad51, which leads to double-strand break repair [226]; cell-cycle regulation by inducing G0-G1 arrest, probably as a result of cyclin D1 downregulation [227]. In addition, injection of PTEN into isolated nuclei enhances apoptosis-induced DNA fragmentation [228]. Recently, Song et al. have also revealed that nuclear PTEN interacts with the anaphase-promoting complex (APC) and enhances its stability and tumour-suppressive activity, in a phosphatase-independent manner [229].

Notably, even if in cells the vast majority of PTEN appears to exist as a monomer and usually only a small percentage of cellular PTEN stably binds to a particular associating factor, PTEN can also be recruited to special plasma membrane locations through binding of its PDZ-interaction motif with PDZ-containing membrane-anchored proteins [230] including MAGI-2 [231], PAR-3 [232], the microtubule-associated serine/threonine kinases MAST-1 and MAST-3 [233], and NHERF [234]. A recent proteomics study suggests that there are additional PTEN-interacting proteins [235], remarkably PP2a, which attracted our attention and is also addressed in this paper. Some PTEN-binding proteins might simply act as an adaptor protein to guide PTEN to specific cellular compartments and subsequently to function there. Although the physiological relevance of many of these interactions needs to be validated, it is possible that some of these associating proteins can regulate PTEN function, including its enzymatic activity; conversely, it is also likely that PTEN regulates the function of its binding partners [236].

Other interesting studies carried out by Liu et al., using fluorescence recovery after photobleaching (FRAP) of GFP-PTEN, revealed that nuclear PTEN had a very rapid diffusion and appeared not to be tethered. Conversely, cytoplasmic PTEN diffused more slowly, suggesting that there are transient interactions with immobile cytoplasmic structures [237]. The apparent tethering of PTEN to cytoplasmic structures raises an intriguing question. Does tethering enhance PTEN activity by placing PTEN close to substrate or does it prevent PTEN from acting at the plasma membrane? [201]. Data regarding PTEN's subcellular localization could lead to new insights, especially considering the recent work of Zhu et al., demonstrating a mitochondrial location of PTEN and its crucial role as mediator of apoptosis [238].

It is possible that a subpopulation of cellular PTEN is dedicated to (and sufficient for) a specific biological function and that there are specific regulatory mechanisms defining this subpopulation. Research on these regulatory mechanisms should provide exciting avenues of investigation and may identify additional functions of PTEN that depend on its subcellular localization. These should help us further elucidate the importance of this protein in human health and disease and—given the distinct localization of PTEN in normal and cancerous cells—might identify potential therapeutic targets.

## 6. Protein Kinase C

PKC comprises a multigene family of related serine/threonine kinases that involve a lot of signal transduction pathways, for example, cell proliferation, differentiation, apoptosis, and autophagy [239]. PKCs exhibit a high molecular heterogeneity, occurring in at least 10 different isoforms differing in biochemical properties and sensitivity to activators. PKCs are lipid-sensitive enzymes that are activated by growth factor receptors that stimulate phospholipase C (PLC), the enzyme that generates diacylglycerol (DAG) and inositol trisphosphate (IP3), with the latter being involved in the mobilization of intracellular Ca^2+^. There are also pharmacological activators of PKC, such as phorbol 12-myristate 13-acetate (PMA). PMA exerts its function by anchoring PKCs in their irreversibly active conformations to the plasma membrane [240]. PKC isozymes are classified as Ca^2+^-dependent, Ca^2+^-independent, and atypical, according to their sensitivity to Ca^2+^ and DAG. For their activation, the conventional PKC (cPKCs: *α*, *β*I, *β*II, *γ*) require additional free Ca^2+^ and DAG. The novel PKCs (nPKCs: *δ*, *ε*, *η*, *θ*) are activated by DAG; the atypical PKCs (aPKCs: *ζ*, *λ*/*ι*) are independent of Ca^2+^ and DAG [241].

**Figure 2:**
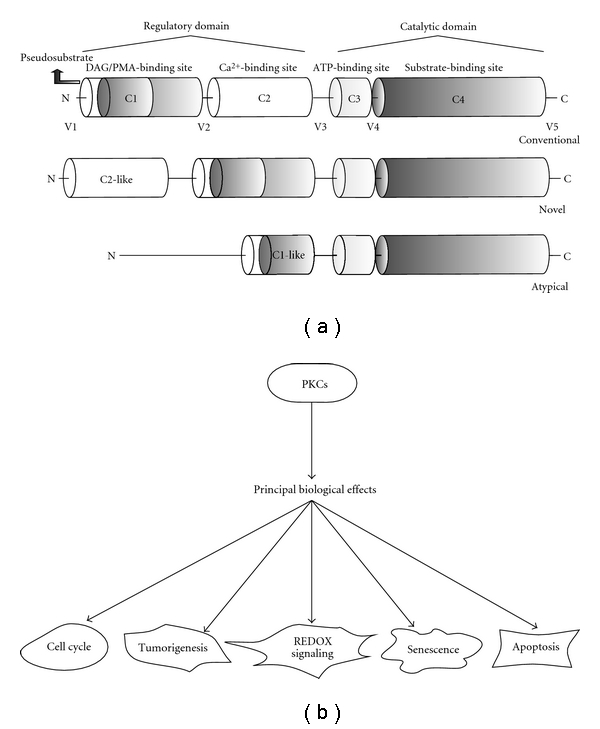
(a) Domain structure of protein kinase C (PKC) isoforms. All PKC family members comprise four conserved domains (C1–4) and five variable (V) domains. All isoforms contain a pseudosubstrate domain (PS) that maintains PKC in a catalytically inactive form. The C1 domains are the molecular sensors of phorbol 12-myristate 13-acetate (PMA)/diacylglycerol (DAG) in cPKC and nPKC isoforms. Atypical PKCs have only one C1 domain and are unable to bind DAG. The C2 domains function as calcium-dependent phospholipid binding modules in cPKCs, whereas nPKC C2 domains do not bind calcium, and aPKCs are lacking these domains. (b) PKCs signalling. The diagram summarizes current knowledge of the PKCs signalling pathways. Full and further details are provided in the text.

The PKC polypeptide consists of a C-terminal catalytic domain and an N-terminal regulatory domain that are separated by a flexible hinge region [242]. cPKCs contain four homologous domains (C1, C2, C3, and C4) interspaced by isozyme-unique variable domains (V1, V2, V3, V4, and V5). The C1 region is a putative membrane-binding domain, the C2 region appears to be related to the Ca^2+^ sensitivity of the enzyme, the C3 region contains the catalytic site and the ATP-binding site, and the C4 region appears to be necessary for recognition of the substrate to be phosphorylated. In nPKCs, there is no C2 homologous domain (and thus, they are insensitive to Ca^2+^); aPKCs lack both the C2 and one-half of the C1 homologous domain (and thus, they are insensitive to both DAG and Ca^2+^) (Figure 2(a)) [243, 244]. In PKCs there are three constitutively phosphorylated sites in the kinase domain. These phosphorylations occur on the activation loop site and two C-terminal sites (TM) [245]. The maturation of PKCs occurs through ordered and constitutive phosphorylations that are very important for their stability and catalytic activity [246]. The first phosphorylation event (by PDK1) is controlled by the conformation of PKC, which is in an open conformation; the pseudosubstrate is removed from the substrate-binding cavity, and the activation loop site is free to be phosphorylated by PDK1 [247]. The phosphorylation of the TM, which depends on mTORC2 [245], stabilizes the structure of mature PKC by anchoring the C-terminal tail on the upper lobe of the kinase [245, 248].

**Figure 3:**
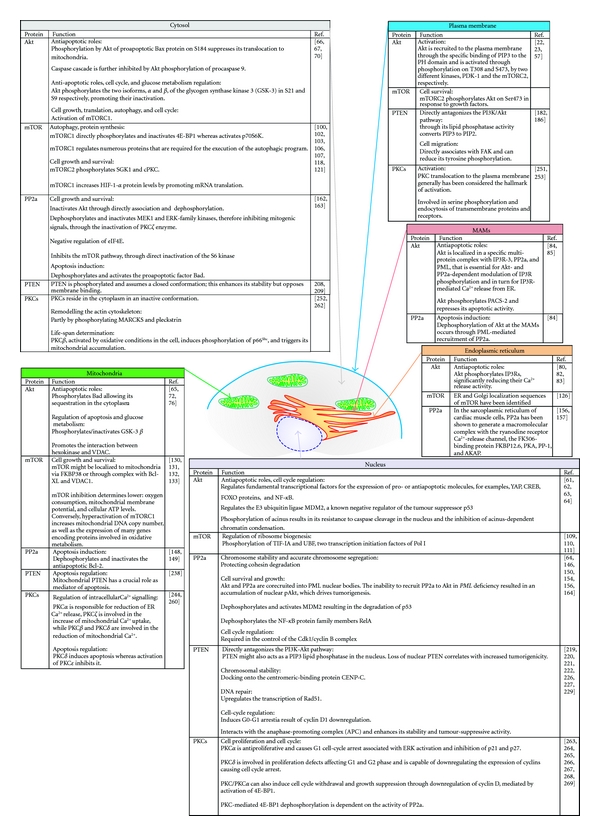
Summary of Akt, mTOR, PP2a, PTEN, and PKC intracellular localization and functions: each table summarized the various physiological processes regulated in the different subcellular compartments. Full and further details are provided in the text.

PKC isoforms are unique, not only with respect to primary structure but also on the basis of expression patterns, subcellular localization, activation in vitro, and responsiveness to extracellular signals. PKC isozymes are homologous enzymes in which their subcellular localization determines the functional specificity. The localization facilitates crosstalk between different signalling intermediates, targeted substrate phosphorylation, and regulation of catalytic activity [239]. PKCs are thought to reside in the cytoplasm in an inactive conformation and, after cell activation, these proteins translocate to the plasma membrane, cytoplasmic organelles, or nucleus (Figure 3) [249, 250]. Translocation of PKC*α*, *β*, *δ*, *ε*, and *ζ* to mitochondria, Golgi, nuclear, or perinuclear regions results in regulation of mitosis, cell survival pathways, and apoptosis [251]. In the cytosol, PKCs interact with several different proteins, including receptors for activated C kinase (RACKS), the product of the par-4 gene, zeta-interacting protein, lambda-interacting protein, and AKAPs. PKCs are also involved in remodelling the actin cytoskeleton, partly by phosphorylating specific PKC substrates such as the myristoylated alanine-rich PKC substrate (MARCKS) protein and pleckstrin. As such, PKCs influence many distinct aspects of cell function [252]. PKC translocation to the plasma membrane generally has been considered the hallmark of activation (and frequently has been used as a measure of PKC isoform activation in cells). PKC activation in the plasma membrane results in serine phosphorylation and endocytosis of transmembrane proteins and receptors [251]. PKC isoforms also translocate to specialized membrane compartments such as lipid rafts or caveolae [253]. A sizeable body of evidence collected over the last 20 years has also shown PKC to be capable of translocating to the nucleus. Furthermore, PKC isoforms are resident within the nucleus. Studies from independent laboratories have led to the identification of quite few nuclear proteins which are PKC substrates and to the characterization of nuclear PKC-binding proteins which may be critical for fine-tuning PKC function in this cell microenvironment. Several lines of evidence suggest that nuclear PKC isozymes are involved in the regulation of biological processes as important as cell proliferation and differentiation, gene expression, neoplastic transformation, and apoptosis (interested readers should refer to [249, 254]). As mentioned above, certain PKCs also accumulate in mitochondria. Nearly 15 years ago, the *α* and *β* isoforms of PKC were detected in a subset of mitochondria in carp retinal Müller cells [255]. These immunoelectron microscopy studies showed that the kinase was localized in the inner membrane. Since then, several data have confirmed that PKC isoforms play a direct role in regulating mitochondrial function. In particular, two isoforms of nPKCs, PKC*δ*, and PKC*ε* show opposite effects on apoptosis: activation of PKC*δ* induces and/or enhances the apoptotic events that occur during ischemia-reperfusion and malignant progression of cancer cells, whereas activation of PKC*ε* inhibits and/or reduces these events (recently reviewed in [244]).

Mitochondria mediate diverse cellular functions including energy generation and intracellular signalling through the generation of ROS (reactive oxygen species) and regulation of intracellular Ca^2+^ signalling [244, 256]. Changes in the spatial distribution and concentration of Ca^2+^ in the cytoplasm constitute a very important intracellular signalling pathway in cellular physiology, including cell proliferation [257]. Ca^2+^ is an activator of some PKCs, and at the same time PKC-dependent phosphorylation reactions change the spatiotemporal pattern of cellular Ca^2+^ responses. PKC isoforms are involved in decoding high- and low-frequency Ca^2+^ spiking [258] and also in shaping the Ca^2+^ signals and Ca^2+^ release from the ER after agonist stimulation [259, 260]. In particular, PKC*α* is responsible for reduction of ER Ca^2+^ release, PKC*ζ* is involved in the increase of mitochondrial Ca^2+^ uptake, while PKC*β* and PKC*δ* are involved in the reduction of mitochondrial Ca^2+^ uptake in HeLa cells [260]. In addition, signalling by PKCs has been shown to encompass a remarkable complexity during redox stress, with differences among PKC isoforms also belonging to the same subgroup [250]. Intracellular redox state is routinely subjected to modifications during cell life, and redox stress may be involved in the pathogenesis of several diseases [244]. PKCs contain, both in the N-terminal regulatory domain and in the C-terminal catalytic domain, regions that are susceptible to redox modifications. Redox stresses change the phosphorylation state of PKC and in turn may regulate their activity; hence, PKCs are proposed to be activated in oxidative stress conditions [261]. Recently, we demonstrated that PKC*β*, activated by oxidative conditions in the cell, induced phosphorylation of p66^Shc^ (a protein that translates oxidative damage into cell death, since it acts as producer of ROS within mitochondria) and triggered mitochondrial accumulation of the protein after it is recognized by the prolyl isomerase Pin1 [262]. 

As mentioned previously, PKCs are also involved in cellular proliferation, in particular during the G1 and the G2/M phases of cell cycle. PKC*α* is antiproliferative and causes G1 cell-cycle arrest associated with ERK activation and inhibition of p21 and p27 in the intestinal epithelial cells [263]. PKC*δ* is involved in proliferation defects affecting G1 and G2 phases [264], and is capable of downregulating the expression of cyclins causing cell-cycle arrest in lung adenocarcinoma cells [265, 266]. PKC/PKC*α* can also induce cell cycle withdrawal and growth suppression in intestinal epithelial cells, an event associated with rapid downregulation of cyclin D1 [267, 268], mediated by activation of 4E-BP1 and inhibition of cap-dependent translation initiation [269]. PKC signalling has been shown to promote dephosphorylation of Thr45 and Ser64 on 4E-BP1, residues directly involved in its association with eIF4E, and further studies have indicated that PKC-mediated 4E-BP1 hypophosphorylation is dependent on the activity of PP2a [39].

The PKC family has been implicated in several pathologies as neoplastic transformation, ageing, diabetes, and angiogenesis. Several studies indicate that increased PKC levels can be found in malignant transformation in breast [270], lung, and gastric carcinomas [271] and in disease progression. PKCs have distinct and opposing roles in cancer development. For example, PKC*ε* overexpression has proliferative properties in rat fibroblasts [272] and has been shown to be upregulated in various types of cancer [273], while PKC*α* and PKC*δ* are downregulated [274, 275]. In fact, PKC*δ* is antiproliferative, and these effects are tissue-dependent [266, 276]. The levels of PKC*β*II are relevant in colonic epithelial cell proliferation and progression to colon carcinogenesis. Increased expression of PKC*β*II was observed in both aberrant crypt foci (ACF; preneoplastic lesions of the colon) and colon tumours, as compared to normal colonic epithelium [244]. There is also a correlation between changes in PKC expression and disease progression. 

In conclusion, PKCs are involved in a wide array of cellular pathways (Figure 2(b)) and potentially represent a good biological target in the treatment of disease. For example, assuming that redox stresses change the phosphorylation state and thus the activity of PKC, a potential therapeutic strategy could be the modulation of PKC activity using antioxidant agents to prevent ROS concentrations in cancer, since the formation of free radicals appears to have an important role in tumour progression. There are several cellular nonenzymatic antioxidants, such as glutathione, thiols, vitamins and metals, isoflavones, polyphenols and flavanoids. Antioxidants alone, however, are not specific and must necessarily be associated with drugs specific for different isoforms [244].

## 7. Conclusions and Perspectives

This paper describes the functional relationship between protein kinases and protein phosphatases in regulating signalling transduction pathways in eukaryotic cells, with a particular focus on cell cycle and apoptosis. The ability to balance a complex network of signal transduction pathways is a key element in homeostasis, enabling a cell to react accurately to external stimuli by proliferating, differentiating, or even dying. The balance between prosurvival and proapoptotic signalling is very sophisticated and the integration of the complex web of incoming signals will ultimately determine cell proliferation or cell death. One of the main mechanisms by which a normal cell appropriately and promptly transduces signals is the reversible and dynamic process of protein phosphorylation [277]. The multiple positive and negative interactions, and the subsequent cellular responses are further modulated by regulated localization of different signalling molecules to key intracellular locations, to facilitate exquisite control of function. Disturbing of this complex equilibrium results in the deregulation of a plethora of cellular processes and subsequent development of diseases. Indeed, the majority of oncogenes identified thus far encode protein kinases, and dysregulation in their activity is required for cancer initiation and maintenance. Although abundant evidence supports the role of kinase oncogenes in cancer development, the study of protein phosphatases and their regulation has only recently become an expanding field of research aimed at shedding light on the importance of these proteins in cancer. Specific protein phosphatases may also play a key role in cancer progression by acting as important regulators of kinase activation and the phosphorylation status of proteins involved in signal transduction [166, 196]. 

Both protein phosphatases and protein kinases play a role in modulating cell responses, yielding a wide array of potential pharmaceutical targets in cancer research. Towards these proteins, it is possible to develop small molecules that specifically inhibit or activate any abnormal cell responses that lead to cell proliferation or cell death [266, 278]. Indeed, several targeted therapies focused on inhibiting particular kinases have now been approved for clinical use, while an increasing number of studies are aimed at identifying new targets and strategies based on the counterbalancing activation of phosphatases.

## Abbreviations

4E-BP1: Eukaryotic initiation factor 4E binding protein-1
ACF: Aberrant crypt foci
Acinus: Apoptotic chromatin condensation inducer in the nucleus
AGC: cAMP-dependent, cGMP-dependent, and protein kinase C
AKAP: A-kinase anchoring protein
AMPK: 5′ AMP-activated protein kinase
APC: Anaphase-promoting complex
Asp: Aspartate
ATG: Autophagy-related
ATP: Adenosine triphosphate
Bad: Bcl-2-associated death promoter
Bax: Bcl-2-associated X protein
BTK: Bruton's tyrosine kinase
Bcl-2: B-cell lymphoma 2
Ca^2+^: Calcium ions
CaMKIV: CaM-dependent kinase IV
Cdk: Cyclin-dependent kinases
CK2: Casein kinase 2
CREB: cAMP response element-binding
DAG: Diacylglycerol
DAPP1: Dual adapter for phosphotyrosine and 3-phosphotyrosine and 3-phosphoinositide
eIF4: Eukaryotic initiation factor 4
ER: Endoplasmic reticulum
ERK: Extracellular signal-regulated kinase
FAK: Focal adhesion kinase
FAT: FRAP-ataxia-telangiectasia
FATC: FAT carboxy-terminal
FIP200: Focal adhesion kinase family interacting protein of 200 kD
FKBP12: FK506-binding protein 12 kDa
FOXO: Forkhead box O
FRAP: Fluorescence recovery after photobleaching
FRB: FKPB12-rapamycin binding
GAP: GTPase-activating protein
GRP1: General receptor for phosphoinositides-1
GSK-3: Glycogen synthase kinase 3
HIF: Hypoxia-inducible factor
HK: Hexokinase
IGF-1: Insulin-like growth factor 1
IP3: Inositol 1,4,5-trisphosphate
IP3R: Inositol 1,4,5-trisphosphate receptor
LCMT1: Leucine carboxyl methyltransferase 1
MAMs: Mitochondrial-associated membranes
MAPK: Mitogen-activated protein kinase
MARCKS: Myristoylated alanine-rich PKC substrate
MAST 1/3: Microtubule-associated serine/threonine kinases MAST-1 and MAST-3
MDM2: Murine double minute 2
MMAC: Mutated in multiple advanced cancers
NF-*κ*B: Nuclear factor kappa-light-chain-enhancer of activated B cells
p70S6K: Ribosomal p70S6 kinase
PACS-2: Phosphofurin acidic cluster sorting protein-2
pAkt: Phosphorylated Akt
PDK1: Phosphoinositide-dependent kinase-1
PDZ: Postsynaptic density protein—*Drosophila* disc large tumour suppressor—zonula occludens 1 protein
PH: Pleckstrin homology
PI3K: Phosphoinositide 3-kinase
Pin1: Prolyl isomerase
PIP2: Phosphatidylinositol 4,5-bisphosphate
PIP3: Phosphatidylinositol 3,4,5-trisphosphate
PKA: Protein kinase A
PKB: Protein kinase B
PKC: Protein kinase C
aPKC: Atypical PKC
cPKC: Conventional PKC
nPKC: Novel PKC
PLC: Phospholipase C
PMA: Phorbol 12-myristate 13-acetate
PML: Promyelocytic leukemia protein
PP1: Protein phosphatase 1
PP2a: Protein phosphatase 2a
PRAS40: Proline-rich Akt substrate of 40 kDa
PRK2: PKC-related kinase 2
PTEN: Phosphatase and tensin homolog deleted on chromosome 10
RACKS: Receptors for activated C kinase
Raptor: Regulatory associated protein of mTOR
RHEB: Ras homologue enriched in brain
Rictor: Rapamycin-insensitive companion of mTOR
ROS: Reactive oxygen species
Ser: Serine
SGK1: Serum/glucocorticoid regulated kinase 1
Sin1: Stress-activated protein kinase-interacting protein
STAT: Signal transducer and activator of transcription
TEP1: Transforming growth factor-*β*-regulated and epithelial-cell-enriched phosphatase 1
Thr: Threonine
TM: Turn motif
mTOR: Mammalian target of rapamycin
mTORC1/2: Mammalian target of rapamycin complex 1/2
TSC1/2: Tuberous sclerosis complex 1/2
VDAC: Voltage-dependent anion channel
YAP: Yes-associated protein.
